# Heterogeneity in pulmonary emphysema: Analysis of CT attenuation using Gaussian mixture model

**DOI:** 10.1371/journal.pone.0192892

**Published:** 2018-02-14

**Authors:** Mizuho Nishio, Yutaka Tanaka

**Affiliations:** 1 Clinical PET Center, Institute of Biomedical Research and Innovation, Minatojimaminamimachi, Chuo-ku, Kobe, Hyogo, Japan; 2 Department of Diagnostic Imaging and Nuclear Medicine, Kyoto University Graduate School of Medicine, 54 Kawahara-cho, Shogoin, Sakyo-ku, Kyoto, Kyoto, Japan; 3 Preemptive Medicine and Lifestyle Disease Research Center, Kyoto University Hospital, 53 Kawahara-cho, Shogoin, Sakyo-ku, Kyoto, Kyoto, Japan; 4 Department of Radiology, Chibune General Hospital, Tsukuda, Nishi-Yodogawa-ku, Osaka, Osaka, Japan; Forschungszentrum Borstel Leibniz-Zentrum fur Medizin und Biowissenschaften, GERMANY

## Abstract

**Purpose:**

To utilize Gaussian mixture model (GMM) for the quantification of chronic obstructive pulmonary disease (COPD) and to evaluate the combined use of multiple types of quantification.

**Materials and methods:**

Eighty-seven patients (67 men, 20 women; age, 67.4 ± 11.0 years) who had undergone computed tomography (CT) and pulmonary function test (PFT) were included. The heterogeneity of CT attenuation in emphysema (HC) was obtained by analyzing a distribution of CT attenuation with GMM. The percentages of low-attenuation volume in the lungs (LAV), wall area of bronchi (WA), and the cross-sectional area of small pulmonary vessels (CSA) were also calculated. The relationships between COPD quantifications and the PFT results were evaluated by Pearson’s correlation coefficients and through linear models, with the best models selected using Akaike information criterion (AIC).

**Results:**

The correlation coefficients with FEV_1_ were as follows: LAV, −0.505; HC, −0.277; CSA, 0.384; WA, –0.196. The correlation coefficients with FEV_1_/FVC were: LAV, –0.640; HC, –0.136; CSA, 0.288; WA, –0.131. For predicting FEV_1_, the smallest AIC values were obtained in the model with LAV, HC, CSA, and WA. For predicting FEV_1_/FVC, the smallest AIC values were obtained in the model with LAV and HC. In both models, the coefficient of HC was statistically significant (*P*-values = 0.000880 and 0.0441 for FEV_1_ and FEV_1_/FVC, respectively).

**Conclusion:**

GMM was applied to COPD quantification. The results of this study show that COPD severity was associated with HC. In addition, it is shown that the combined use of multiple types of quantification made the evaluation of COPD severity more reliable.

## Introduction

Chronic obstructive pulmonary disease (COPD) is characterized by chronic airflow limitation, which is usually progressive and not fully reversible [1]. COPD can lead to irreversible structural changes such as remodeling of airways and destruction of lung parenchyma. These structural changes are caused by abnormal inflammatory response toward cigarette smoke or other noxious gases. COPD is in effect a syndrome, with elements of bronchitis, airway hyperreactivity, inflammation, and emphysema in variable proportions [2].

Computed tomography (CT) and computer software made it possible to quantitatively evaluate the structural changes in the lungs caused by COPD, and quantitative evaluation of CT was more sensitive than visual assessment for evaluating emphysema [3]. Although clinical evaluation of CT images is usually qualitative or semi-quantitative, the qualitative or semi-quantitative evaluation of COPD has suffered from inter-observer variability [4]. Quantitative evaluation of CT images has the potential to identify phenotypes of COPD and assess the progression of COPD.

The most widely used method to quantify emphysema on CT is the percentage of low-attenuation volume in lungs (LAV) [5]. However, no single type of quantification can guarantee an accurate assessment of COPD severity. There is, therefore, a need for a new way to quantify COPD. Many types of COPD quantification have been suggested in previous studies: LAV and D (D was obtained by analyzing the size distribution of low-attenuation lung regions) for emphysema [4, 6]; the percentage of wall area (WA) for airway wall change [7, 8]; the percentage of the cross-sectional area of small pulmonary vessels (CSA) for vascular alteration [9]; and Patlak analysis of 18F-fluorodeoxyglucose positron emission tomography for the inflammatory state [10]. Combining these methods, such as a combination of LAV and WA, has been investigated and shown to be superior to using a single type of quantification [7, 11–14].

We hypothesized that heterogeneity of CT attenuation was useful for quantifying COPD. Although spatial heterogeneity of emphysema was investigated in previous studies [15, 16], here we focused on the heterogeneity of CT attenuation. To assess this, we used Gaussian mixture model (GMM). In GMM, the distribution of CT attenuation is approximated by a mixture of Gaussian distributions for which the mean and variance can be calculated. Because variance reflects the heterogeneity of a Gaussian distribution, the heterogeneity of CT attenuation can be calculated by GMM. In addition, we evaluated combinations of multiple types of quantifications, in contrast to the previous studies, which mainly investigated the combined use of just two types. We speculated that the severity of COPD could be assessed more accurately by the use of multiple types of quantification.

In summary, the aims of the current study were: i) to validate GMM for COPD quantification by analyzing CT attenuation distribution in the lungs, ii) to assess whether the heterogeneity of CT attenuation obtained from GMM was useful for COPD quantification, and iii) to evaluate the combined use of LAV, CSA, WA, and heterogeneity of CT attenuation.

## Materials and methods

This retrospective study was approved by the institutional review boards of Institute of Biomedical Research and Innovation and Chibune General Hospital. The acquisition of informed consent was waived by the review boards.

### Patients

Patients who visited our institution because of their respiratory symptom (such as chronic cough and dyspnea) were examined retrospectively. If the patient underwent CT and pulmonary function test (PFT) and the interval between CT and PFT was less than 90 days, the patient was included in the current study. COPD was diagnosed based on the Global Initiative for Chronic Obstructive Lung Disease criteria [1]. These patients had no exacerbation at the CT and PFT examinations.

This study included 87 consecutive patients (67 men, 20 women; age, 67.4 ± 11.0 years). 39 patients were diagnosed with COPD; 38 were smokers without COPD; and 10 were non-smokers. The mean smoking history of all the 87 patients, the 39 patients with COPD, and the 38 smokers without COPD were 45.8 ± 38.63, 58.3 ± 45.8, and 45.1 ± 24.1 pack-years, respectively. The mean interval between CT and PFT was 17.3 ± 39.1 days.

### CT scan

Noncontrast helical CT scans were acquired from the lung apices through the lung bases with a 320-detector-row scanner (Aquilion ONE; Toshiba Medical Systems, Otawara, Japan) by using automated exposure control. The scan parameters were as follows: noise index, 10; tube current, 200 ± 66.5 mA; tube potential, 120 kV; gantry rotation time, 0.35 s in one patient, 0.6 s in two patients, and 0.5 s in all the other patients. After receiving careful instruction about breathing, the patients were scanned in the supine position during a deep inspiratory breath hold. To reduce computational cost of GMM, raw CT data were reconstructed into 5-mm-thick images with soft tissue kernel (FC 13 or 14). The CT scanner was calibrated regularly.

### Pulmonary function test

The PFT was performed with an automated spirometer (HI-801 or CHESTAC-8900, CHEST M.I., INC., Tokyo, Japan). Vital capacity, forced expiratory volume in one second (FEV_1_), forced vital capacity (FVC), and the ratio of forced expiratory volume in one second to forced vital capacity (FEV_1_/FVC), were obtained. Apart from FEV_1_/FVC, these were expressed as percentages of the standard predicted values.

### Image preprocessing

The acquired CT images were processed by our prototype software. First, the lungs were automatically segmented from the CT images using region growing, an auto-detected seed point, and a threshold at −500 HU.

The CT attenuation (HU) of all the lung voxels were collected, and the mean, variance, skewness, and kurtosis of the CT attenuation distribution were calculated to examine the distribution of lung voxels. Then, using GMM, the distribution of CT attenuation was approximated by a mixture of Gaussian distributions, and the mean and variance of each distribution was calculated. In GMM, the distribution is approximated by:

$$\sum_{i=1}^{K}\gamma_{i}\;N(\mu_{i},\sigma_{i}^{2}),$$

where, *K* is the number of Gaussian distributions determined experimentally, *N(μ*_*i*_*, $\sigma_{i}^{2}$)* is a Gaussian distribution with mean *μ*_*i*_ and variance $\sigma_{i}^{2}$, and *γ*_*i*_ gives the relative weightings of the distributions and satisfies $\sum_{i=1}^{K}\gamma_{i}=1,\gamma_{i}\geq 0\;(i=1,2,3,\ldots ,K)$. *K* = 2, 3, 4, 5, 6, 7, and 8 were tested in the current study, and *K* = 4 was selected based on results of preliminary experiments (for the preliminary experiments, please refer to Tables B and C in S1 File of Supporting information). When a larger *K* was used, the computational cost of GMM was unacceptable. The mean *μ*_*i*_ and variance $\sigma_{i}^{2}$ (*i =* 1, 2, 3, …, *K*) obtained by GMM were sorted by the value of *μ*_*i*_. As a result, the variance $\sigma_{1}^{2}$ corresponded to the lowest mean *μ*_*1*_. The mean *μ*_*1*_ and variance $\sigma_{1}^{2}$ were used for the detailed statistical analysis ($\sigma_{1}^{2}$ was referred to as HC in the current study). Here, dimensions of *μ*_*1*_ and HC obtained by GMM were HU and HU^2^, respectively. Python (version 2.7; http://www.python.org/) and scikit-learn (version 0.17.1; http://scikit-learn.org/) were used for performing GMM.

LAV was obtained as the percentage of the number of low-attenuation lung voxels to the total number of lung voxels [5]. In the current study, 5 different thresholds were evaluated, and −970 HU was selected as the threshold of LAV (for the results of 5 different thresholds, please refer to Table A in S1 File of Supporting information). CSA values were calculated by applying several modifications to the method described in the previous study [9]. First, python-2.7 and the OpenCV package for python were used for blob detection. Second, CT images covering the whole chest were analyzed using segmented lungs. Third, the calculation of CSA was fully automatic. Last, the slice thickness of CT images differed from that of the previous study. Because of these differences, multiple thresholds of CT attenuation and other CSA parameters were tested, and the optimal combination of the parameters was selected (in Table D in S1 File of Supporting information, the effect of CSA parameters was shown). The optimal parameters were as follows: threshold of CT attenuation, −730 HU; range of circularity, 0.9–1.0; size of vessel area, 5–10 mm^2^. Measurement of the airway wall change was performed using AirwayInspector, which is available at http://airwayinspector.acil-bwh.org/ and was used for the previous study [17, 18]. In each patient, the fourth generation of bronchi at RB1, LB1+2, and RB10 were selected by a consensus reading of two board-certified radiologists (MN and YT). The software detected the inner and outer boundaries of the airway wall at the selected bronchi, and WA was calculated automatically. The mean value of WA across the three bronchi was used for the statistical analysis.

### Statistical analysis

To test whether the quantification reflected the severity of COPD, Pearson's correlation coefficients were calculated between the results of quantification and PFT. Correlation was also evaluated between LAV and *μ*_*1*_ and between LAV and mean of the CT attenuation distribution.

Next, linear models were used to investigate the relationship between the PFT results and the COPD quantification. One set of linear models was built to predict FEV_1_ using the COPD quantification, and another set was built to predict FEV_1_/FVC. In each set, combinations of LAV, CSA, WA, and HC were used as predictor variables. Because the values of predictor variables were not normally distributed, log transformation was applied to the predictor variables. The coefficients of the predictor variables were evaluated with their *P*-values, and the best models were selected as those with the lowest values based on Akaike information criterion values (AIC) [19]. According to the previous studies, the difference in AIC of more than 1 or 2 was regarded as significant [20, 21]. All analyses were performed using R-3.1.0 (available at http://www.R-project.org/). *P*-values less than 0.05 were considered statistically significant.

## Results

**Fig 1:**
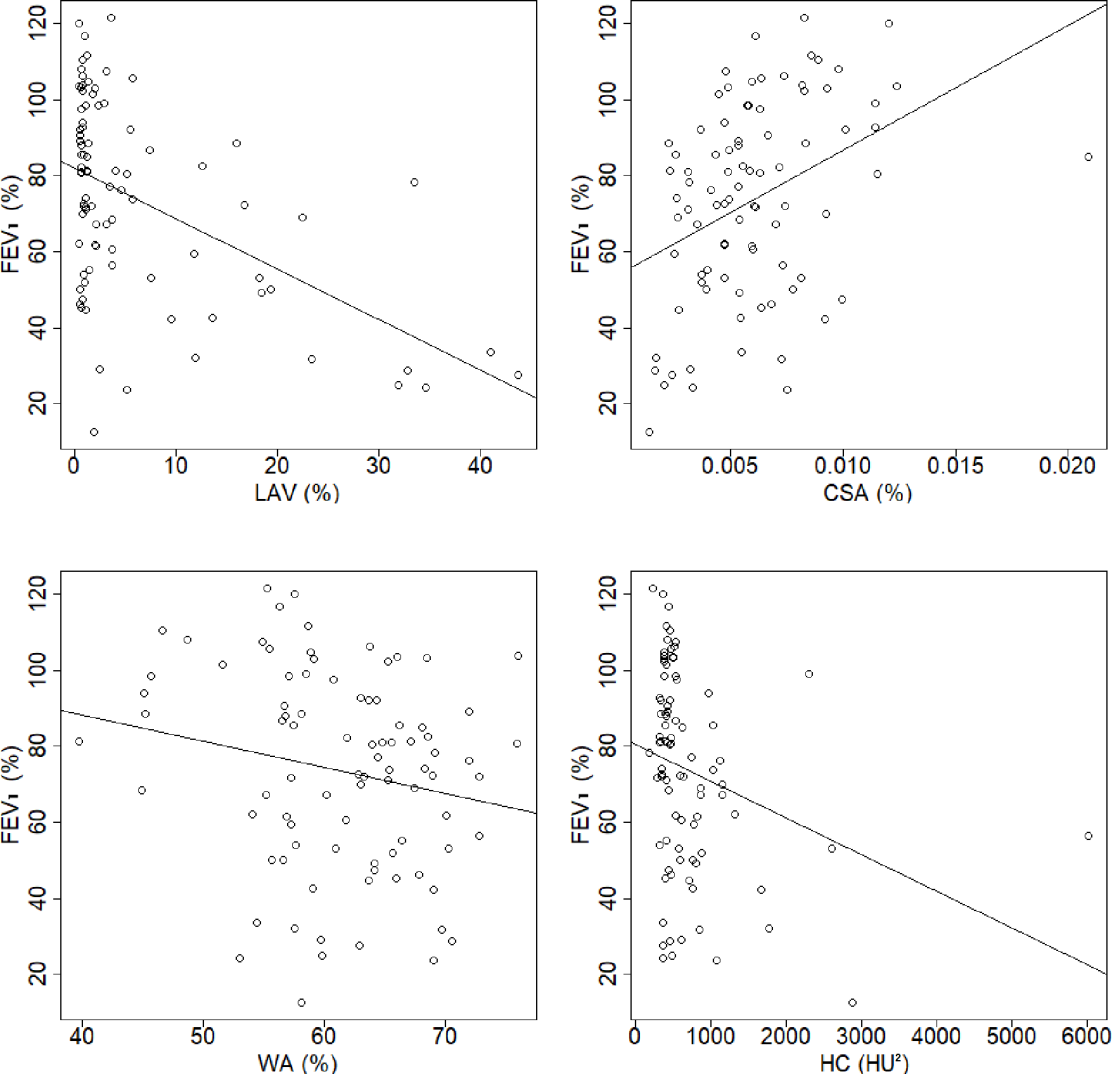
Scatter plots of FEV_1_ against COPD quantification. A)–D) show the plots for LAV, CSA, WA, and HC, respectively. Abbreviations: FEV_1_, forced expiratory volume in one second; LAV, percentage of low-attenuation volume in the lungs; HC, heterogeneity of CT attenuation in emphysema; CSA, percentage of cross-sectional area for small pulmonary vessels; WA, percentage of wall area.

**Fig 2:**
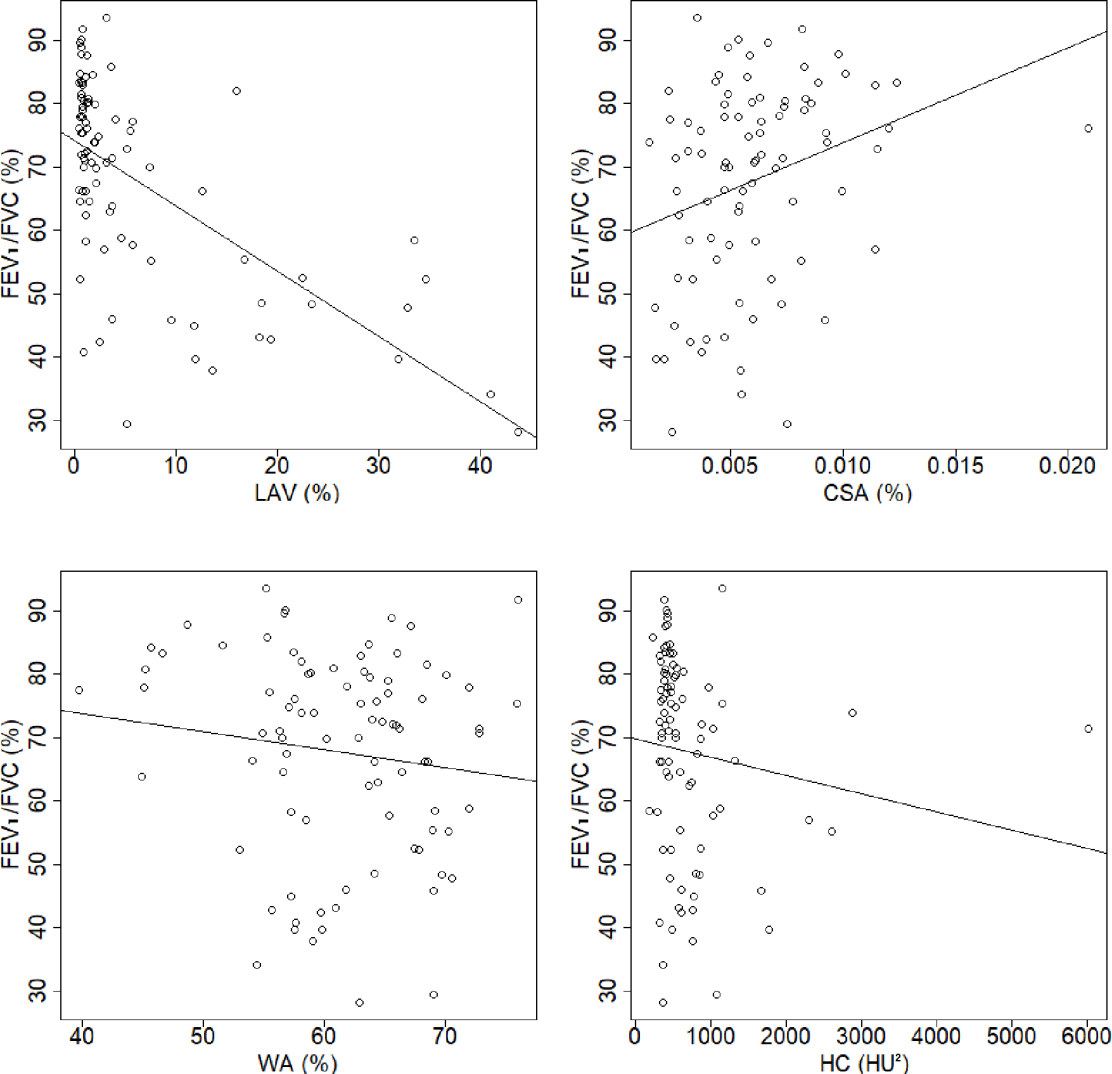
Scatter plots of FEV_1_/FVC against COPD quantification. A)–D) show the plots for LAV, CSA, WA, and HC, respectively. Abbreviations: FEV_1_/FVC, ratio of forced expiratory volume in one second to forced vital capacity; LAV, percentage of low-attenuation volume in the lungs; HC, heterogeneity of CT attenuation in emphysema; CSA, percentage of cross-sectional area for small pulmonary vessels; WA, percentage of wall area.

**Fig 3:**
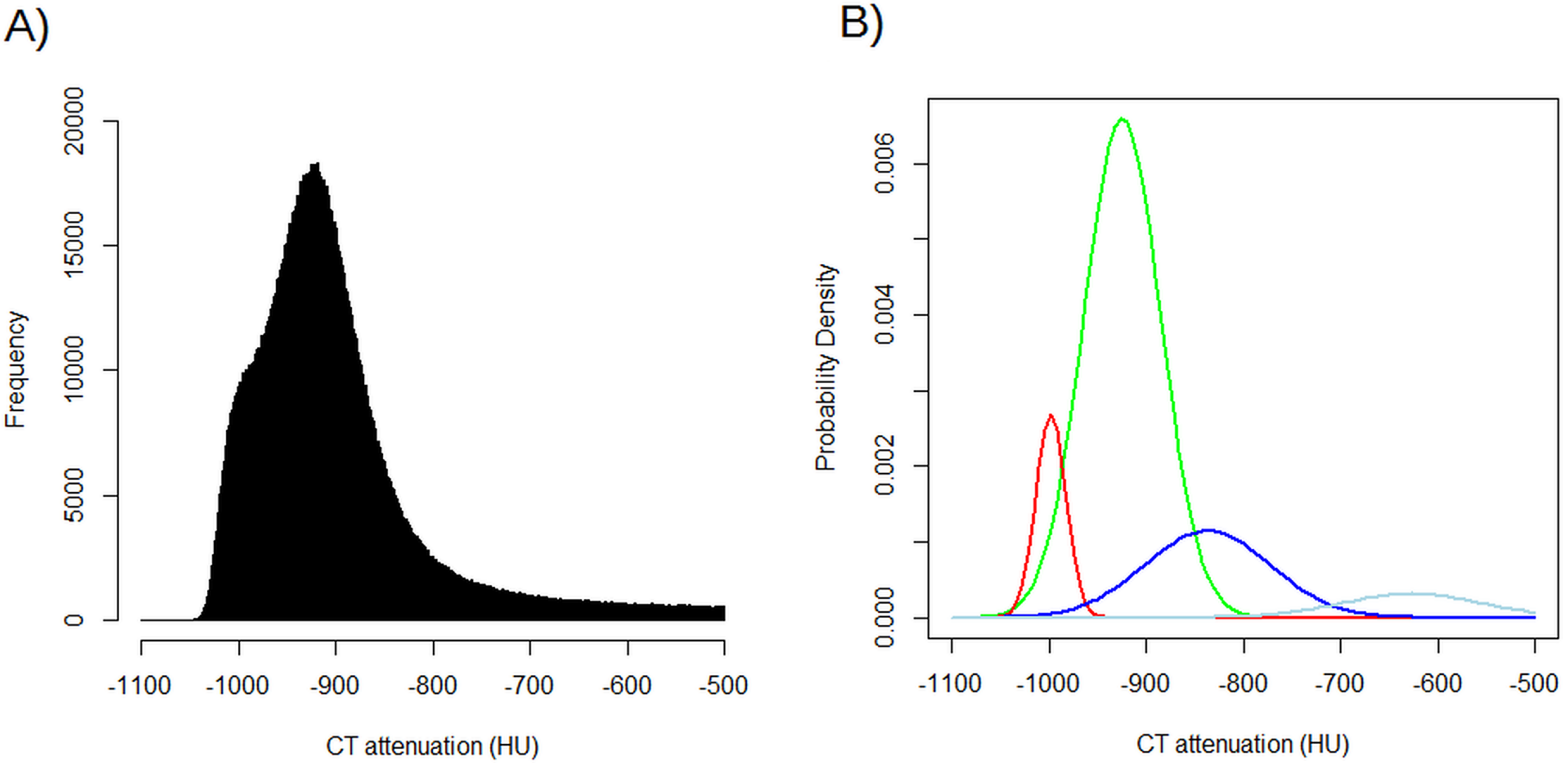
Histogram of CT attenuation for lungs and result of GMM in 59-year-old man with COPD. A) shows the histograms of CT attenuation for lungs when width of histogram bar was 1 HU. B) shows the four Gaussian distributions obtained by GMM. The mixture of these four Gaussian distributions approximated the histogram. The Gaussian distribution with the lowest mean is represented by the red solid line, which corresponds to the distribution of emphysema. Abbreviations: GMM, Guassian mixture model; COPD, chronic obstructive pulmonary disease.

Patient characteristics and the results of the PFT and COPD quantification values are summarized in Table 1. Table 2 shows the correlations coefficients between PFT results and COPD quantification values. Figs 1 and 2 show scatter plots of FEV_1_ and FEV_1_/FVC, respectively, against the COPD quantification values. Fig 3 shows the schematic illustration of histograms of CT attenuation in lungs and the Gaussian distributions obtained by GMM.

**Table 1:** Summary of patient characteristics, PFT results, COPD quantification values.

	All		Non-smoker		Smoker without COPD		COPD	
Variables	Mean	SD	Mean	SD	Mean	SD	Mean	SD
N	87		10		38		39	
Age (year)	67.4	10.98	67.5	14.03	65.71	11.44	69.03	9.66
Sex = M (number of male)	67		1		33		33	
Smoking history (pack year)	45.8	38.63	0	0	45.05	24.06	58.29	45.83
FVC (%)	91.17	23.93	98.82	19.24	93.98	25.33	86.47	23.19
FEV_1_/FVC (%)	67.74	15.95	81.97	5.19	78.87	6.15	53.25	11.80
FEV_1_ (%)	73.54	25.96	99.61	12.53	85.41	21.33	55.29	19.56
VC (%)	97.12	23.68	98.32	24.34	100.85	24.16	93.18	23.03
Mean of CT attenuation distribution (HU)	−862.48	35.11	−839.63	34.54	−850.8	32.08	−879.7	30.47
Variance of CT attenuation distribution (HU^2^)	8100	1920	7260	1490	7990	2180	8420	1690
Skewness of CT attenuation distribution	1.81	0.49	1.82	0.47	1.77	0.58	1.83	0.39
Kurtosis of CT attenuation distribution	3.78	2.06	3.73	1.74	3.69	2.45	3.88	1.74
LAV (%)	6.22	9.89	0.72	0.30	2.11	2.71	11.64	12.62
*μ*_*1*_ (HU)	−917.8	38.06	−890.3	25.10	−904.9	28.38	−937.3	39.87
HC (HU^2^)	702.2	748.4	483.4	175.1	697.3	992.1	763.1	534.3
CSA (%)	0.00599	0.00304	0.00817	0.00265	0.00635	0.00348	0.00507	0.00229
WA (%)	61.37	7.38	62.07	10.5	60.33	7.68	62.21	6.14

Note: The PFT results were expressed as percentages of the standard predicted values, apart from FEV_1_/FVC. Abbreviations: CSA, percentage of cross-sectional area for small pulmonary vessels; FEV_1_, forced expiratory volume in one second; FEV_1_/FVC, ratio of forced expiratory volume in one second to forced vital capacity; FVC, forced vital capacity; HC, heterogeneity of CT attenuation in emphysema; LAV, percentage of low-attenuation volume in lungs; PFT, pulmonary function test; VC, vital capacity. WA, percentage of wall area; *μ*_*1*_, the lowest mean from the Gaussian mixture model.

**Table 2:** Pearson’s correlation coefficients between the quantitative evaluation of COPD and PFT results.

Variables	FEV_1_	FEV_1_/FVC
Mean of CT attenuation distribution (HU)	0.194	0.513
Variance of CT attenuation distribution (HU^2^)	−0.300	−0.222
Skewness of CT attenuation distribution	0.184	−0.039
Kurtosis of CT attenuation distribution	0.167	−0.031
LAV (%)	−0.505	−0.640
*μ*_*1*_ (HU)	0.292	0.554
HC (HU^2^)	−0.277	−0.136
CSA (%)	0.384	0.288
WA (%)	−0.196	−0.131

Abbreviations: CSA, percentage of cross-sectional area for small pulmonary vessels; FEV_1_, forced expiratory volume in one second; FEV_1_/FVC, ratio of forced expiratory volume in one second to forced vital capacity; HC, heterogeneity of CT attenuation in emphysema; LAV, percentage of low-attenuation volume in lungs; WA, percentage of wall area; *μ*_*1*_, the lowest mean from the Gaussian mixture model.

Table 2 showed that LAV, the variance of the distribution, and CSA had relatively strong correlations with FEV_1_. LAV, *μ*_*1*_, and the mean of the distribution had relatively strong correlations with FEV_1_/FVC. The correlation coefficient between LAV and *μ*_*1*_ and that between LAV and the mean of the distribution were −0.811 and −0.629, respectively. These results suggest that LAV, *μ*_*1*_, and the mean of the distribution were related to the severity of emphysema.

Tables 3 and 4 show the results of the linear models, and AIC values of all the models using LAV, HC, CSA and WA were shown in Tables E and F in S1 File of Supporting information. In Table 3, the models with HC had more accurate predictability than those without HC. This means that heterogeneity of CT attenuation in emphysema was independently useful for quantifying COPD severity. Model 4 in Table 3 (with predictor variables LAV, HC, CSA, and WA) had the smallest AIC among the models examined in this study, and this model was the best among those in Table 3 and Table E in S1 File of Supporting information. Table 4 shows that the smallest AIC was obtained in Model 2, which included LAV and HC as predictor variables. Table F in S1 File of Supporting information shows that the difference of AIC values between the model with LAV and HC and that with LAV, HC, and CSA was small, which means that there was not one best model. However, for the models to predict FEV_1_/FVC, combining LAV and HC was better than LAV alone.

**Table 3:** Results of the linear model between FEV_1_ and the COPD quantification.

Model index	Predictor variable	Coefficient	*P*-value	AIC of model
1				794.8
	LAV	−9.42	1.58 x 10^−6^	
2				785.2
	LAV	−8.63	3.33 x 10^−6^	
	HC	−13.7	0.000871	
3				776.6
	LAV	−6.14	0.00104	
	HC	−13.9	0.000385	
	CSA	15.6	0.00152	
4				774.6
	LAV	−5.95	0.00126	
	HC	−12.9	0.000880	
	CSA	16.3	0.000791	
	WA	−33.8	0.0532	

Note: Log transformation was applied to values of predictor variables. Abbreviations: AIC, Akaike information criterion value; CSA, percentage of cross-sectional area for small pulmonary vessels; FEV_1_, forced expiratory volume in one second; HC, heterogeneity of CT attenuation in emphysema; LAV, percentage of low-attenuation volume in the lungs; WA, percentage of wall area.

**Table 4:** Results of the linear model between FEV_1_/FVC and the COPD quantification.

Model index	Predictor variable	Coefficient	*P*-value	AIC of model
1				680.4
	LAV	−8.02	6.24 x 10^−13^	
2				678.2
	LAV	−7.77	1.61 x 10^−12^	
	HC	−4.39	0.0441	
3				679.9
	LAV	−7.52	1.93 x 10^−10^	
	HC	−4.41	0.0441	
	CSA	1.53	0.577	
4				680.2
	LAV	−7.45	2.65 x 10^−10^	
	HC	−4.03	0.0670	
	CSA	1.81	0.509	
	WA	−12.5	0.214	

Note: Log transformation was applied to values of predictor variables. Abbreviations: AIC, Akaike information criterion value; CSA, percentage of cross-sectional area for small pulmonary vessels; FEV_1_/FVC, ratio of forced expiratory volume in one second to forced vital capacity; HC, heterogeneity of CT attenuation in emphysema; LAV, percentage of low-attenuation volume in the lungs; WA, percentage of wall area.

## Discussion

The current study demonstrated three main points: i) GMM could be used for quantifying the severity of COPD; ii) the heterogeneity of CT attenuation in emphysema obtained from GMM was useful for quantifying COPD; and iii) Combination of COPD quantification values allowed COPD severity to be evaluated accurately.

To our knowledge, this was the first study to apply GMM to COPD quantification. Previously, GMM has been used for several biomedical or medical applications [22, 23]. We hypothesized that the distribution of CT attenuation consisted of multiple components, and that these components could be captured separately as Gaussian distributions by using GMM. In this study, we examined the relationship between emphysema quantification (LAV) and the Gaussian distribution with the lowest mean (*μ*_*1*_) obtained with GMM. The correlation between LAV and *μ*_*1*_ was strong (correlation coefficient = −0.811); hence, this suggests that the Gaussian distribution with the lowest mean *μ*_*1*_ corresponded to the emphysema component. This result supports our hypothesis.

We also hypothesized that the heterogeneity of CT attenuation (HC) was useful for COPD quantification. Because the Gaussian distribution with the lowest mean *μ*_*1*_ corresponded to the distribution of emphysema, the variance of the Gaussian distribution with the lowest mean *μ*_*1*_ reflected the heterogeneity of CT attenuation in emphysema. Table 2 shows that HC was negatively correlated with FEV_1_, and Tables 3 and 4 show that HC was independently useful for COPD quantification, which verify our hypothesis. The previous studies showed that the spatial distribution of emphysema was associated with COPD severity [15, 16]. In these studies, LAV was used to assess the spatial distribution of emphysema. Because the CT attenuation of lung voxels was binarized in LAV, the distribution of CT attenuation could not be assessed using LAV. Our study investigated the distribution of CT attenuation in emphysema, and the heterogeneous distribution of emphysema was associated with low FEV_1_.

While the Gaussian distribution with the lowest mean was investigated intensively, the other Gaussian distributions were not examined in the current study. Both COPD and interstitial lung abnormality were caused by smoking [17]. Interstitial lung abnormality was represented as relatively high density area, such as ground-glass opacity. We speculated that, using GMM, the distribution of lung voxels in COPD patients might be divided into distributions of normal lung tissue, emphysema, and interstitial lung abnormality. Therefore, it may be possible to use GMM for the assessment of interstitial lung abnormality and emphysema separately. However, because it was difficult to quantify normal lung tissue and interstitial lung abnormality automatically, we focused on the Gaussian distribution with the lowest mean in the current study.

The current study investigated the combined use of four types of COPD quantification (LAV, HC, CSA, and WA). AIC values in Tables 3 and 4 and those in Tables E and F in S1 File of Supporting information show that the model with four types of quantification was the best for the prediction of FEV_1_, and that, for the prediction of FEV_1_/FVC, the model with LAV and HC was better than that with LAV alone. The results of these linear models showed that LAV and HC were independently useful for the COPD quantification. As shown in the previous studies, LAV has been most widely used for emphysema quantification. In accordance with these results, LAV was the strongest predictor in the linear models of our study. Our results also showed that HC was significant predictor, supporting our hypothesis that heterogeneity of CT attenuation in emphysema was associated with severity of COPD.

As shown in Table 2, the correlations between WA and FEV_1_ and between WA and FEV_1_/FVC were relatively weak (coefficients = −0.196 and −0.131, respectively). Nakano et al. suggested that measurements of large airway wall thickening could be used for COPD quantification [7]. However, the study of Lee et al. failed to show a direct relationship between the severity of PFT abnormality and WA [8]. Our results were intermediate between those of the previous two studies. Although WA was measured by the consensus reading of the two radiologists with the aid of AirwayInspector, we speculate that measurement error caused by technical problems related to WA weakened the correlation between WA and the PFT results. Population differences may be attributable to changes in correlation between WA and PFT results; Nakano et al. included all smokers [7], Lee et al. included patients with moderate or severe COPD [8], and we included non-smokers, smokers without COPD, and COPD patients. In addition, the location where WA was measured affected the correlations between WA and FEV_1_ and between WA and FEV_1_/FVC, because airflow limitation in COPD was more closely related to WA in distal airway than that in proximal airways [24].

There are several limitations in the current study. First, this study was performed retrospectively, and the number of patients included in this study was relatively small. To confirm our results, it will be necessary to use a large cohort of patients as a prospective study. Second, the CT parameters used in this study were different from those commonly used in previous studies; for example, use of automated exposure control and the thickness of CT images (thickness = 5 mm) might affect our results for COPD quantification. Third, although the relationship between results of PFT and COPD quantification was investigated in the current study, those with clinical outcomes, health status, and disease progression of COPD were not examined. Because FEV_1_ correlated weakly with clinical outcomes and health status in COPD patients [25], other types of metric should be used when comparing COPD quantification with clinical outcomes or disease progression. We will perform a prospective study for investigating whether results of GMM are correlated well with these factors. Last, we did not evaluate the effect of cluster analysis. In a previous study [20], the usefulness of combined use of LAV and D was examined for predicting PFT results. It is difficult to precisely compare the results between the previous study and the current study because of the difference in study design. However, the improvement of statistical model obtained by addition of D seems to be smaller than by addition of HC based on the values of AIC. Therefore, it is speculated that the usefulness of D would be limited in the current study.

In conclusion, our results showed that GMM could be applied to COPD quantification, and that COPD severity was associated with the heterogeneity of CT attenuation in emphysema. In addition, combining COPD quantification values, including the heterogeneity of CT attenuation in emphysema, improved the reliability of COPD severity evaluation.

## Supporting information

S1 File **S1 File including -Tables A-F.** According to Table A, the best correlation of LAV was obtained when -970 HU was used. According to Tables B and C, *K* = 4 was selected when LAV and HC were combined. According to Table D, the optimal parameters of CSA were as follows: threshold of CT attenuation, −730 HU; range of circularity, 0.9–1.0; size of vessel area, 5–10 mm^2^. Tables E and F show AIC results of all the model predicting FEV_1_ and FEV_1_/FVC, respectively.
(DOCX)

S2 File Scatter plots of PFT results against COPD quantification after outlier removal. Figure A shows scatter plots of FEV_1_ against COPD quantification after outlier removal. Figure B shows scatter plots of FEV_1_/FVC against COPD quantification after outlier removal.
(DOCX)

S3 File **S3 File including Figure A and Table A.** Figure A shows plots of regression model diagnostics for the linear model between FEV_1_ and the COPD quantification in Model 4 of Table 3. Table A shows results of the linear model between FEV_1_ and the COPD quantification after removal of 6 data points.
(DOCX)

S4 File **S4 File including Figure A and Table A.** Figure A shows plots of regression model diagnostics for the linear model between FEV_1_/FVC and the COPD quantification in Model 2 of Table 4. Table A shows results of the linear model between FEV_1_/FVC and the COPD quantification after removal of 5 data points.
(DOCX)

S5 File Speculation regarding Fig 3. Several pixels are less than -1000 HU in Fig 3. S5 File shows speculation for this phenomenon.
(DOCX)
