# Supplementary material for: Heterogeneity in pulmonary emphysema: Analysis of CT attenuation using Gaussian mixture model
Source: PLoS One. 2018 Feb 14;13(2):e0192892. doi: 10.1371/journal.pone.0192892 (PMC5812649; doi:10.1371/journal.pone.0192892)
Supplement: S1 File — including -Tables A-F. According to Table A, the best correlation of LAV was obtained when -970 HU was used. According to Tables B and C, K = 4 was selected when LAV and HC were combined. According to Table D, the optimal parameters of CSA were as follows: threshold of CT attenuation, −730 HU; range of circularity, 0.9–1.0; size of vessel area, 5–10 mm2. Tables E and F show AIC results of all the model predicting FEV1 and FEV1/FVC, respectively. (DOCX) [file pone.0192892.s001.docx]

**S1 File**

**summary**

- According to Table A, the best correlation of LAV was obtained when -970 HU was used.
- According to Tables B and C, *K* = 4 was selected when LAV and HC were combined.
- According to Table D, the optimal parameters of CSA were as follows: threshold of CT attenuation, −730 HU; range of circularity, 0.9–1.0; size of vessel area, 5–10 mm^2^.
- Tables E and F show AIC results of all the model predicting FEV_1_ and FEV_1_/FVC, respectively.

**Table A.**

Pearson’s correlation coefficients between LAV and FEV_1_ at different thresholds. Abbreviations: FEV_1_, forced expiratory volume in one second; LAV, percentage of low-attenuation volume in lungs

| Variables | correlation with FEV_1_ | |
| --- | --- | --- |
| LAV at -910 HU | -0.285 | |
| LAV at -930 HU | -0.397 | |
| LAV at -950 HU | -0.482 | |
| LAV at -970 HU | -0.505 | |
| LAV at -990 HU | -0.453 | |
|  |  |  |

**Table B.**

Pearson’s correlation coefficients between HC and FEV_1_ at different *K*. Abbreviations: FEV_1_, forced expiratory volume in one second; HC, heterogeneity of CT attenuation in emphysema; *K*, number of Gaussian distribution when calculating HC.

| Variables | correlation with FEV_1_ |
| --- | --- |
| HC (*K* = 2) | -0.330 |
| HC (*K* = 3) | -0.248 |
| HC (*K* = 4) | -0.277 |
| HC (*K* = 5) | -0.300 |
| HC (*K* = 6) | -0.299 |
| HC (*K* = 7) | -0.321 |
| HC (*K* = 8) | -0.288 |
|  |  |

**Table C.**

Results of the linear model between FEV_1_ and the COPD quantification. Note: Log transformation was applied to LAV and HC. Abbreviations: FEV_1_, forced expiratory volume in one second; AIC, Akaike information criterion value; LAV, percentage of low-attenuation volume in the lungs; HC, heterogeneity of CT attenuation in emphysema.

| Model index | Predictor variable | AIC of model |
| --- | --- | --- |
| 1 | LAV, HC (*K* = 2) | 793.5 |
| 2 | LAV, HC (*K* = 3) | 792.7 |
| 3 | LAV, HC (*K* = 4) | 785.2 |
| 4 | LAV, HC (*K* = 5) | 786.6 |
| 5 | LAV, HC (*K* = 6) | 786.5 |
| 6 | LAV, HC (*K* = 7) | 785.4 |
| 7 | LAV, HC (*K* = 8) | 785.8 |
|  |  |  |

**Table D.**

Pearson’s correlation coefficients between CSA and FEV_1_ at different parameters. Abbreviations: FEV_1_, forced expiratory volume in one second; CSA, percentage of cross-sectional area for small pulmonary vessels

| CT threshold (HU) | size (mm^2^) | circularity | correlation with FEV_1_ |
| --- | --- | --- | --- |
| -700 | 1–5 | 0.9–1.0 | 0.080 |
| -700 | 5–10 | 0.9–1.0 | 0.333 |
| -710 | 1–5 | 0.9–1.0 | 0.053 |
| -710 | 5–10 | 0.9–1.0 | 0.339 |
| -720 | 1–5 | 0.9–1.0 | 0.097 |
| -720 | 5–10 | 0.9–1.0 | 0.331 |
| -730 | 1–5 | 0.9–1.0 | 0.102 |
| -730 | 5–10 | 0.9–1.0 | 0.384 |
| -740 | 1–5 | 0.9–1.0 | 0.173 |
| -740 | 5–10 | 0.9–1.0 | 0.352 |
| -750 | 1–5 | 0.9–1.0 | 0.170 |
| -750 | 5–10 | 0.9–1.0 | 0.328 |

**Table E.**

AIC results of all the model predicting FEV_1_. Note: Log transformation was applied to values of predictor variables. Abbreviations: AIC, Akaike information criterion value; CSA, percentage of cross-sectional area for small pulmonary vessels; FEV_1_, forced expiratory volume in one second; HC, heterogeneity of CT attenuation in emphysema; LAV, percentage of low-attenuation volume in the lungs; WA, percentage of wall area.

| Predictor Variables | AIC |
| --- | --- |
| No variables | 816.4994 |
| WA | 814.8721 |
| CSA | 798.6065 |
| CSA, WA | 794.6993 |
| HC | 805.7492 |
| HC, WA | 805.448 |
| HC, CSA | 785.9628 |
| HC, CSA, WA | 783.7316 |
| LAV | 794.7768 |
| LAV, WA | 792.941 |
| LAV, CSA | 787.914 |
| LAV, CSA, WA | 784.4465 |
| LAV, HC | 785.229 |
| LAV, HC, WA | 784.6579 |
| LAV, HC, CSA | 776.6248 |
| LAV, HC, CSA, WA | 774.6361 |

**Table F.**

AIC results of all the model predicting FEV_1_/FVC. Note: Log transformation was applied to values of predictor variables. Abbreviations: AIC, Akaike information criterion value; CSA, percentage of cross-sectional area for small pulmonary vessels; FEV_1_/FVC, ratio of forced expiratory volume in one second to forced vital capacity; HC, heterogeneity of CT attenuation in emphysema; LAV, percentage of low-attenuation volume in the lungs; WA, percentage of wall area.

| Predictor Variables | AIC |
| --- | --- |
| No variables | 731.7293 |
| WA | 731.8826 |
| CSA | 724.1578 |
| CSA, WA | 723.5119 |
| HC | 728.2123 |
| HC, WA | 729.0801 |
| HC, CSA | 720.6262 |
| HC, CSA, WA | 720.844 |
| LAV | 680.4444 |
| LAV, WA | 680.2614 |
| LAV, CSA | 682.1676 |
| LAV, CSA, WA | 681.8259 |
| LAV, HC | 678.2212 |
| LAV, HC, WA | 678.71 |
| LAV, HC, CSA | 679.8926 |
| LAV, HC, CSA, WA | 680.2443 |
