# Supplementary material for: Heterogeneity in pulmonary emphysema: Analysis of CT attenuation using Gaussian mixture model
Source: PLoS One. 2018 Feb 14;13(2):e0192892. doi: 10.1371/journal.pone.0192892 (PMC5812649; doi:10.1371/journal.pone.0192892)

**S2 File**

**Figure A. Scatter plots of FEV_1_ against COPD quantification after outlier removal.** A)–D) show the plots for LAV, CSA, WA, and HC, respectively. Note**:** Three data points whose HC was larger than 2500 HU^2^ were removed. Pearson’s correlation coefficients between the quantitative evaluation of COPD and FEV_1_ after outlier removal are as follows: LAV, -0.541; HC, -0.254; CSA, 0.373; WA, -0.196. Abbreviations: FEV_1_, forced expiratory volume in one second; LAV, percentage of low-attenuation volume in the lungs; HC, heterogeneity of CT attenuation in emphysema; CSA, percentage of cross-sectional area for small pulmonary vessels; WA, percentage of wall area.


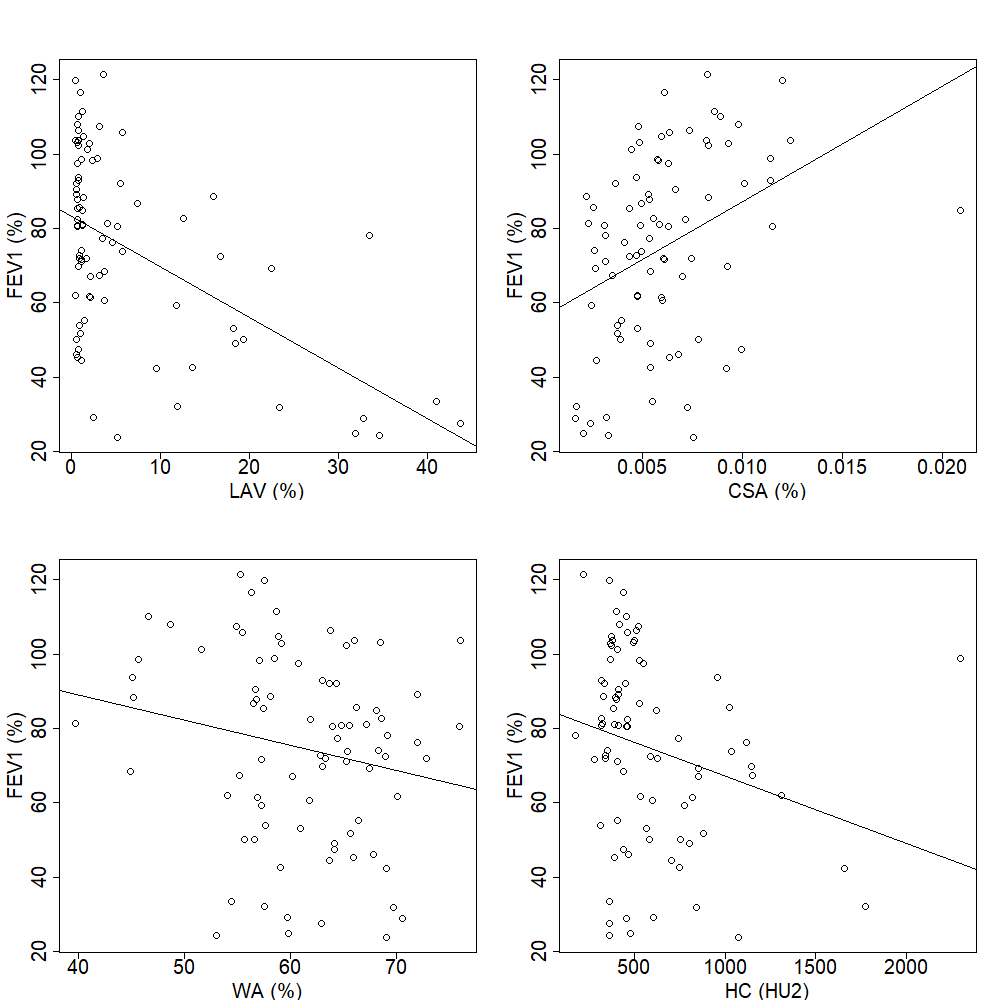


**Figure B.** **Scatter plots of FEV_1_/FVC against COPD quantification after outlier removal.** A)–D) show the plots for LAV, CSA, WA, and HC, respectively. Note**:** Three data points whose HC was larger than 2500 HU^2^ were removed. Pearson’s correlation coefficients between the quantitative evaluation of COPD and FEV_1_/FVC after outlier removal are as follows: LAV, -0.641; HC, -0.313; CSA, 0.307; WA, -0.126. Abbreviations: FEV_1_/FVC, ratio of forced expiratory volume in one second to forced vital capacity; LAV, percentage of low-attenuation volume in the lungs; HC, heterogeneity of CT attenuation in emphysema; CSA, percentage of cross-sectional area for small pulmonary vessels; WA, percentage of wall area.


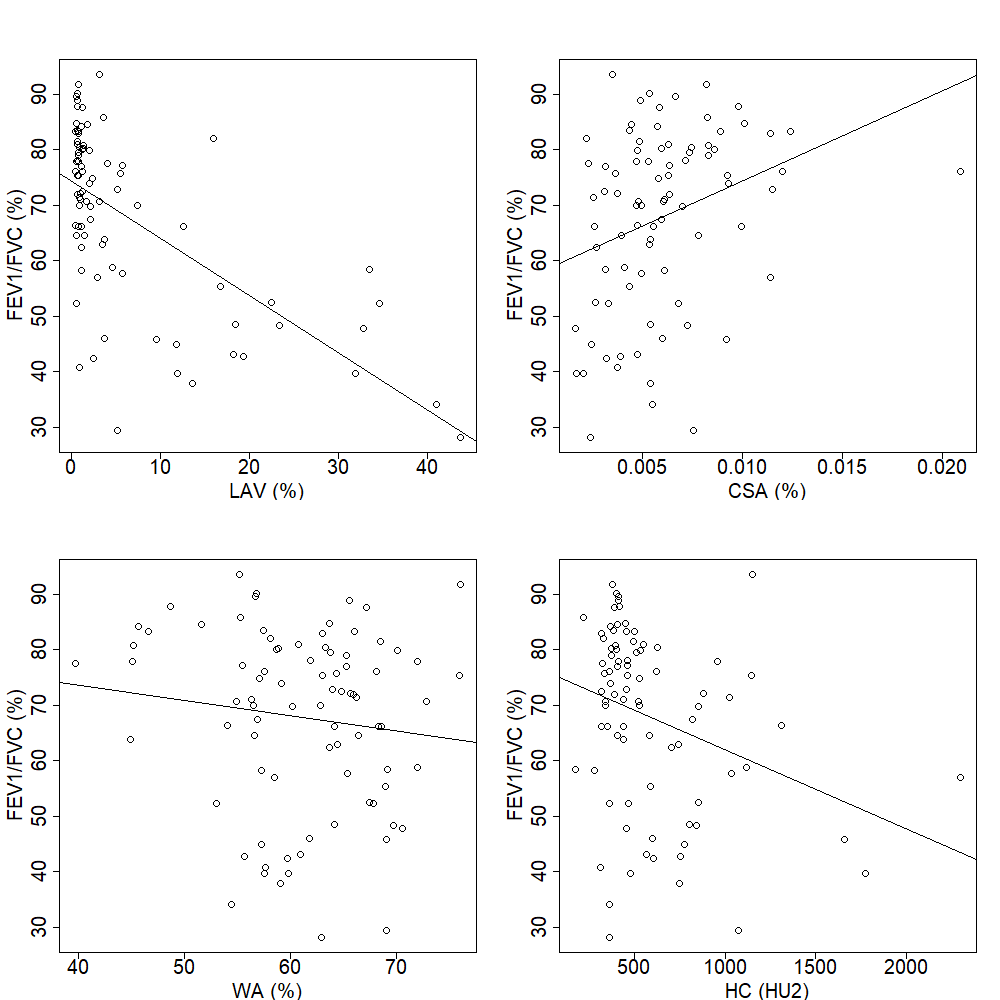

Supplement: S2 File — Figure A shows scatter plots of FEV1 against COPD quantification after outlier removal. Figure B shows scatter plots of FEV1/FVC against COPD quantification after outlier removal. (DOCX) [file pone.0192892.s002.docx]
