# Supplementary material for: Heterogeneity in pulmonary emphysema: Analysis of CT attenuation using Gaussian mixture model
Source: PLoS One. 2018 Feb 14;13(2):e0192892. doi: 10.1371/journal.pone.0192892 (PMC5812649; doi:10.1371/journal.pone.0192892)
Supplement: S3 File — including Figure A and Table A. Figure A shows plots of regression model diagnostics for the linear model between FEV1 and the COPD quantification in Model 4 of Table 3. Table A shows results of the linear model between FEV1 and the COPD quantification after removal of 6 data points. (DOCX) [file pone.0192892.s003.docx]

**S3 File**

**Figure A. Plots of regression model diagnostics for the linear model between FEV_1_ and the COPD quantification in Model 4 of Table 3.** Note: From these plots, 6 data points were identified.


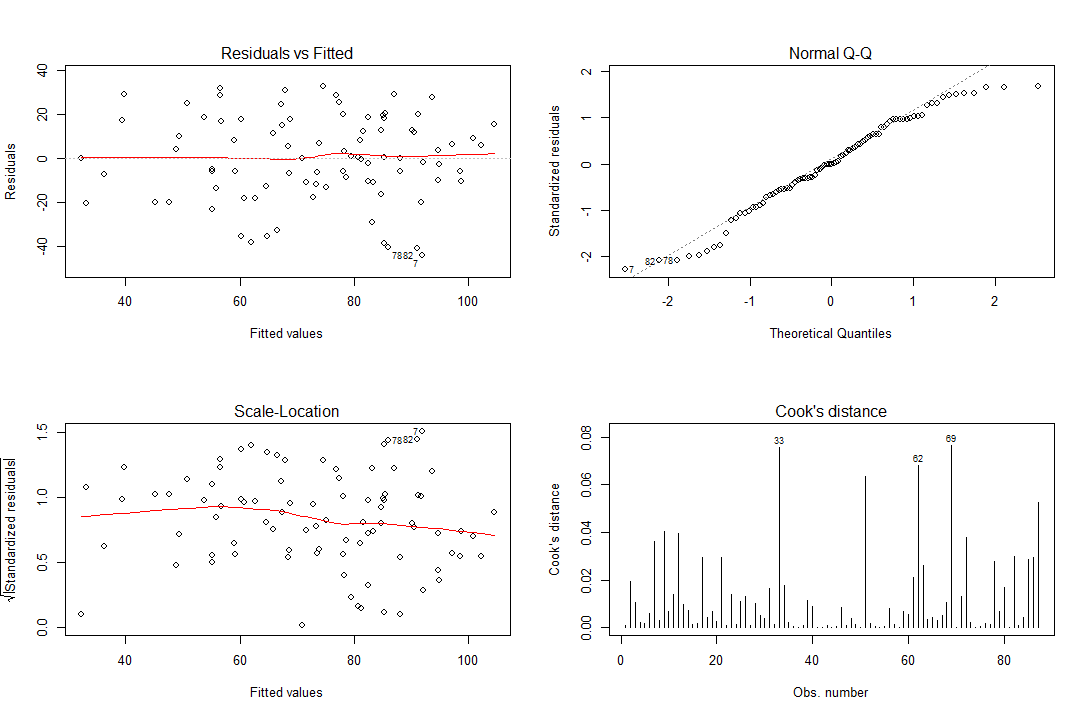


**Table A. Results of the linear model between FEV_1_ and the COPD quantification after removal of 6 data points.**

| Model index | Predictor variable | Coefficient | *P*-value | AIC of model |
| --- | --- | --- | --- | --- |
| 1 |  |  |  | 704.4 |
|  | LAV | −6.56 | 0.000294 |  |
|  | HC | −14.5 | 0.000234 |  |
|  | CSA | 15.7 | 0.00113 |  |
|  | WA | −32.8 | 0.0409 |  |
|  |  |  |  |  |

Note: Model 4 of Table 3 was reevaluated after removal of the 6 data points identified in Figure A. Log transformation was applied to values of predictor variables. Abbreviations: AIC, Akaike information criterion value; CSA, percentage of cross-sectional area for small pulmonary vessels; FEV_1_, forced expiratory volume in one second; HC, heterogeneity of CT attenuation in emphysema; LAV, percentage of low-attenuation volume in the lungs; WA, percentage of wall area.
