# Supplementary material for: Heterogeneity in pulmonary emphysema: Analysis of CT attenuation using Gaussian mixture model
Source: PLoS One. 2018 Feb 14;13(2):e0192892. doi: 10.1371/journal.pone.0192892 (PMC5812649; doi:10.1371/journal.pone.0192892)
Supplement: S4 File — including Figure A and Table A. Figure A shows plots of regression model diagnostics for the linear model between FEV1/FVC and the COPD quantification in Model 2 of Table 4. Table A shows results of the linear model between FEV1/FVC and the COPD quantification after removal of 5 data points. (DOCX) [file pone.0192892.s004.docx]

**S4 File**

**Figure A. Plots of regression model diagnostics for the linear model between FEV_1_/FVC and the COPD quantification in Model 2 of Table 4.** Note: From these plots, 5 data points were identified.


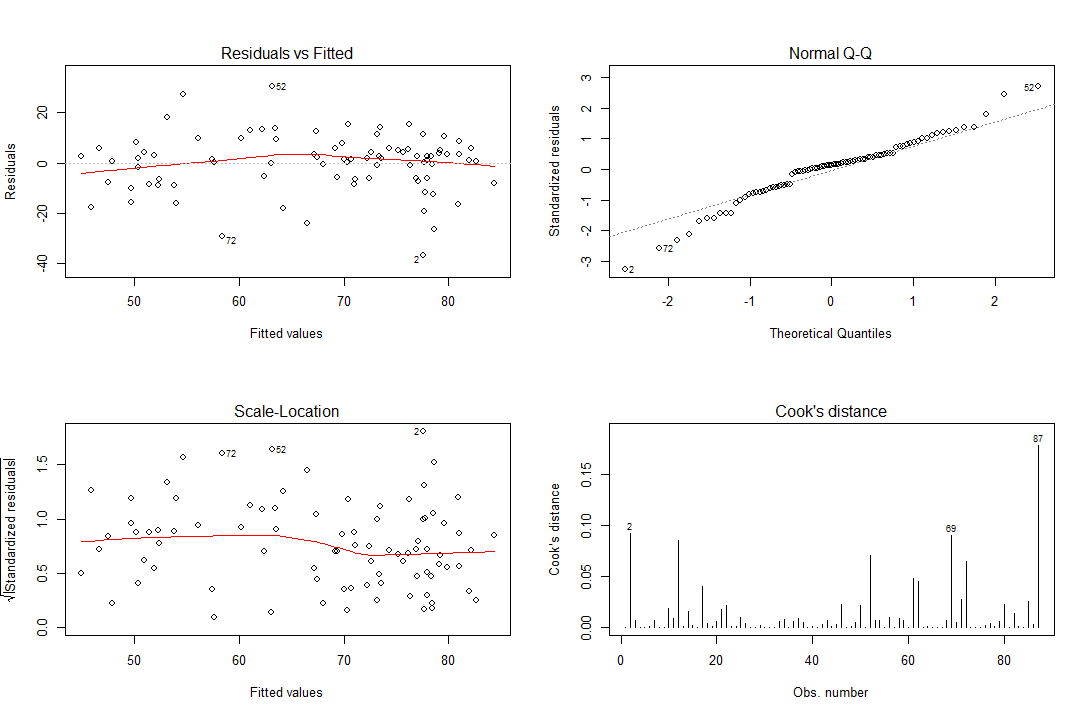


**Table A. Results of the linear model between FEV_1_/FVC and the COPD quantification after removal of 5 data points.**

| Model index | Predictor variable | Coefficient | *P*-value | AIC of model |
| --- | --- | --- | --- | --- |
| 1 |  |  |  | 607.8 |
|  | LAV | −7.76 | 1.17x10^−15^ |  |
|  | HC | −8.78 | 0.000123 |  |
|  |  |  |  |  |

Note: Model 2 of Table 4 was reevaluated after removal of the 5 data points identified in Figure A. Log transformation was applied to values of predictor variables. Abbreviations: AIC, Akaike information criterion value; FEV_1_/FVC, ratio of forced expiratory volume in one second to forced vital capacity; HC, heterogeneity of CT attenuation in emphysema; LAV, percentage of low-attenuation volume in the lungs.
