# Supplementary material for: Heterogeneity in pulmonary emphysema: Analysis of CT attenuation using Gaussian mixture model
Source: PLoS One. 2018 Feb 14;13(2):e0192892. doi: 10.1371/journal.pone.0192892 (PMC5812649; doi:10.1371/journal.pone.0192892)
Supplement: S5 File — Several pixels are less than -1000 HU in Fig 3. S5 File shows speculation for this phenomenon. (DOCX) [file pone.0192892.s005.docx]

**S5 File**

Here, we described our speculation about the presence of voxels less than -1000 HU in Figure 3.

In average, CT values of air are -1000 HU. However, in some scanner models and some scan conditions, the CT value of several voxels can be less than -1000 HU. For example, it is possible to speculate that there are several voxels less than -1000 HU in low-dose CT of Figure 4 of ref. [I]. In addition, Figure 4 of ref. [I] show that noise of low-dose CT makes the CT values of lung voxels widely distributed. Because automated exposure control was used in the current study, it is speculated that the distribution of CT values of lung voxels was affected by noise caused by dose reduction of automated exposure control.

Figure 3B shows that the voxels less than -1000 HU are caused by red line of Figure 3B, which corresponds to emphysema component. The average of this component is about -1000 HU, according to Figure 3B. From these results, we can speculate that, in the case of Figure 3, emphysema component is mainly occupied by air. Thus, this patient was radiologically diagnosed with severe emphysema.

As shown, emphysema component filled with air was affected by noise of dose reduction. Based on Figure 4 of ref. [I], noise makes emphysema component widely distributed. As a result, there are several voxels less than -1000 HU in Figure 3. We speculate that this phenomenon will be seen in the following two conditions: (A) severe emphysema depicted on noisy CT images; and (B) emphysema depicted on very noisy CT images.

**References**

1. Schilham AM, van Ginneken B, Gietema H, Prokop M. Local noise weighted filtering for emphysema scoring of low-dose CT images. IEEE Trans Med Imaging. 2006 Apr;25(4):451–63. doi: 10.1109/TMI.2006.871545.
